# Advancing Workaholism Research

**DOI:** 10.3390/ijerph17249435

**Published:** 2020-12-16

**Authors:** Cristian Balducci, Paola Spagnoli, Malissa Clark

**Affiliations:** 1 Department of Psychology, University of Bologna, 40127 Bologna, Italy; 2 Department of Psychology, University of Campania “Luigi Vanvitelli”, 81100 Caserta, Italy; paola.spagnoli@unicampania.it; 3 Department of Psychology, Franklin College of Arts and Science, University of Georgia, Athens, GA 30602, USA; clarkm@uga.edu

Research on workaholism (also called work addiction by some scholars, especially in the clinical psychology field) has increased substantially in the last few years. A search on PsycInfo using the two terms “workaholism” and “work addiction” covering just the past three years (i.e., January 2018–December 2020) reveals that more than 240 studies have been published on the topic, indicating a widespread interest from researchers all over the world.

Although different definitions of workaholism has been proposed [1,2], researchers are now converging on the idea that workaholism is a significant psychological dysfunction characterized by an irresistible preoccupation for work (i.e., a true obsession) and an uncontrollable internal urge to invest heavily (i.e., time and effort) in work activities. Such work investment is well beyond what is required to meet organizational demands or to reach financial security. According to some, workaholism is a true behavioral addiction [3]. 

It is important to note that, even before the construct of workaholism was introduced, occupational health research had already begun to examine the related constructs of type A behavior pattern and overcommitment. Type A behavior pattern [4] shares with workaholism a strong component of achievement striving in addition to the heavy work investment element. However, type A behavior involves a hostility component which does not seem to be a defining element of workaholism. Perhaps more similar to workaholism is overcommitment [5], of which the main characteristic is an inability to withdraw from work obligations, which is also central to workaholism. Overcommitment may have very detrimental effects on health and well-being outcomes [6,7,8]. Studies in which both overcommitment and workaholism have been measured have found strong correlations between the two [9]. However, a systematic investigation of their common and unique correlates is still lacking in the literature.

Research on workaholism has so far mostly focused on its negative consequences for the affected individuals [1]. Such negative consequences may be identified not only at the psychological level [10] but also at the psychosomatic [11] and physiological [12,13] levels. Additionally, and importantly, the heavy work investment characterizing workaholism does not seem to be associated with higher job performance [14]—although evidence on the relationship between workaholism and objective performance criteria is still sparse. Thus, the available research suggests—with only some exceptions [15]—that workaholism has few advantages for both individuals and organizations, and a number of disadvantages and that it therefore should be prevented as much as possible.

Unfortunately, globalized competition which is the context of the operation of modern organizations has contributed to a widespread long work-hour culture, where workaholic cognition and behavior are constantly reinforced through tangible (e.g., salary, incentives, and promotions) and intangible (e.g., praise) rewards. Additionally, work-related stress, which is a ubiquitous phenomenon [16], is considered an unavoidable part of working life, and organizations openly push workers towards a heavy work investment as necessary for continued employment [17].

Given the above state of affairs, documenting the “individual costs” and the lack of clear-cut organizational advantages of workaholism is important not only to protect workers’ health but also to preserve long-term organizational vitality and productivity. This is the main objective of the present Special Issue on workaholism, collecting a total of 11 contributions from well-known researchers in the field.

The “viewpoint” article by Atroszko and colleagues [18] serves as a good starting point to read the Special Issue contributions. In their paper, Atroszko and colleagues examine the links between work environmental conditions, work addiction, personality disorders and dispositions, and the burnout epidemic as a factor of the global burden of disease.

Four different articles of the Special Issue focus on personality traits and orientations as significant correlates of workaholism. Mazzetti and colleagues [19] explore the role of obsessive–compulsive traits, achievement orientation, perfectionism, and conscientiousness, while Kun et al. [20] focus on low self-esteem and perfectionism. Additionally, Falco at al. [21] investigate the implication of narcissistic tendencies on workaholism (and work engagement) under different conditions defined by the levels of workload, while Avanzi et al. [22] explore the similarities and differences between workaholism and overcommitment. It is clear from the results of these studies that personality traits and orientations, and particularly perfectionistic tendencies, may have something to say when it comes to explaining the genesis of workaholism. Finally, the study by Choi et al. [23] investigates work-related calling as an individual-level characteristic linked to workaholism, specifically exploring the mediating role of obsessive work passion.

Among the different working conditions, workload—as a chronic feature of the job—has received the most attention as a potential antecedent of workaholism. Different studies of the Special Issue further document the role of workload either as a potential main factor in workaholism [19,24] or as a factor interacting with personality traits [25].

Finally, three different studies of the Special Issue further highlight the negative outcomes associated with workaholism by examining employee turnover [26], depression and poor sleep quality [24], and behavioral and emotional problems in children of workaholic parents [27]. On the contrary, the study by Li et al. [28] found that workaholism attenuated the negative relationship between work intensification and well-being. This result was explained by a higher level of job crafting (e.g., seeking resources) carried out by more workaholic individuals. Such findings suggest the need to adopt a more complex approach in future research on workaholism as opposed to focusing on the “main effects” only. Indeed, the study of interactions between workaholism and other variables has been a quite neglected area of research so far [1].

Overall, we believe that the collection of articles included in the present Special Issue provides novel and original evidence on workaholism, furthering our knowledge of the nomological network of the phenomenon.
